# Evolution of microsporidia: An extremely successful group of eukaryotic intracellular parasites

**DOI:** 10.1371/journal.ppat.1008276

**Published:** 2020-02-13

**Authors:** Lina Wadi, Aaron W. Reinke

**Affiliations:** Department of Molecular Genetics, University of Toronto, Toronto, Ontario, Canada; University of Wisconsin Medical School, UNITED STATES

## Overview

Microsporidia are obligate intracellular parasites that can infect a wide range of hosts. They cause death and disease in both humans and agriculturally important animals. They have also become a powerful model for understanding the evolution of intracellular parasites. Recent work has explored how microsporidia genomes have evolved, the evolutionary origins of microsporidia, and how microsporidia adapt to interact with their hosts.

## What do microsporidia have in common?

Microsporidia can only exist outside of their host as environmentally resistant, chitin-containing spores. These spores contain a unique infectious apparatus known as the polar tube. During infection, the polar tube is rapidly discharged and can pierce a host cell, depositing the parasite’s sporoplasm within the host cell [1]. The sporoplasm then proliferates, eventually producing spores that then exit the host to cause subsequent infections. Microsporidia are thought to reproduce mostly asexually, although most are likely diploid based on genomic heterozygosity and conservation of meiotic genes [2–5].

**Fig 1:**
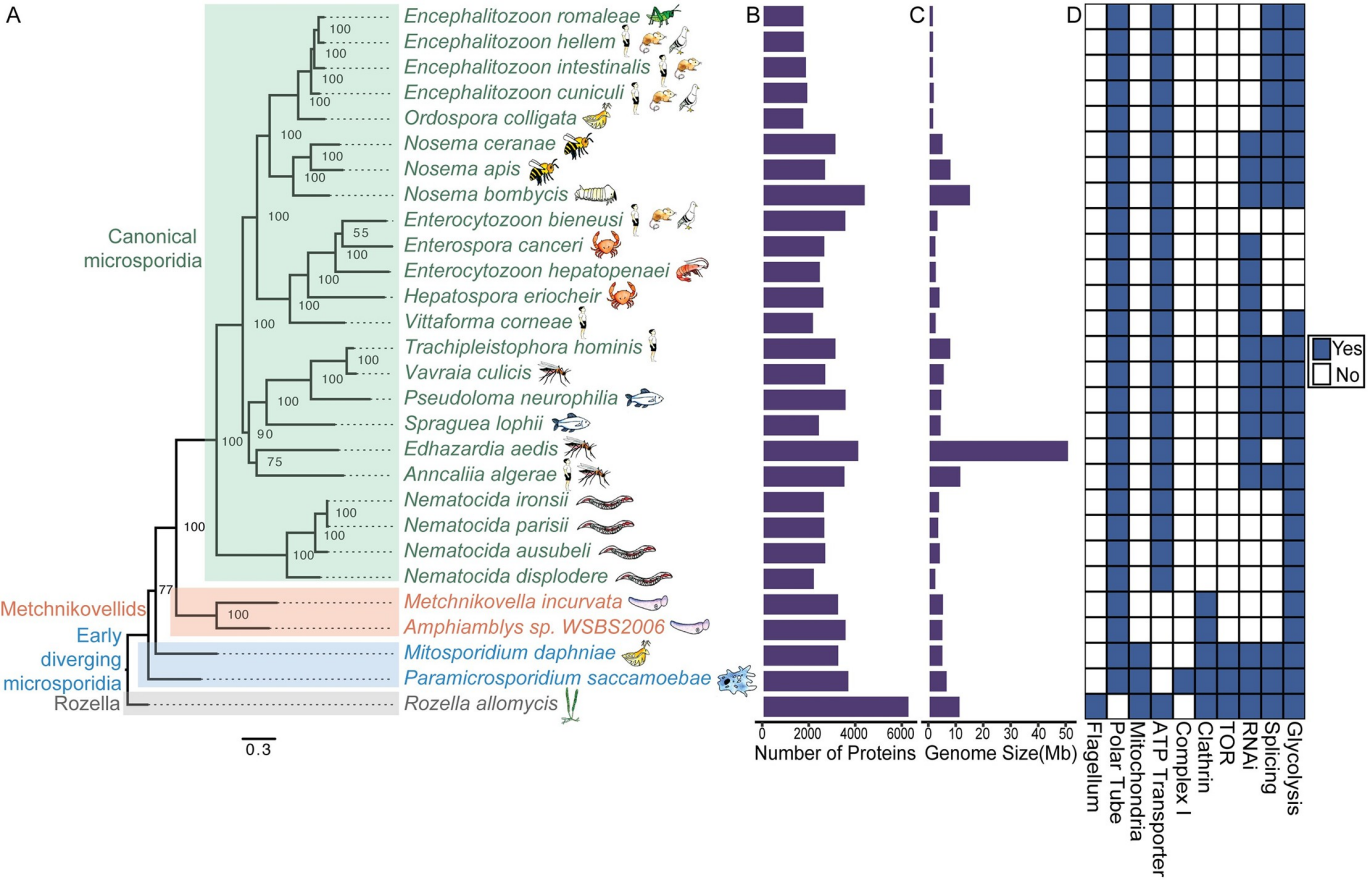
Phylogeny, hosts, and characteristics of microsporidia species. (A) Phylogenetic tree of whole-genome sequenced microsporidia and related species. Tree was generated as described previously using single-copy orthologs present in all species [19,35]. Predicted proteins for each genome were obtained from https://www.ncbi.nlm.nih.gov, except for *Metchnikovella incurvata* and *Enterospora canceri*, which were predicted from genome assemblies using Prodigal 2.6.3. Orthologous gene families were identified using OrthoMCL 2.0.9 using an inflation index of 1.5 and a BLAST E-value cutoff of 10^−5^. Phylogeny was constructed from 43 single-copy orthologs present in all 28 species. Proteins from each orthogroup were aligned using MUSCLE 3.8.31. These alignments were trimmed using trimAl 1.4 with the option-gappyout. Each orthogroup alignment was then concatenated into a single alignment using FASconCAT 1.11. Using ProtTest 3.4.2, it was determined that PROTGAMMAIWAGF was the best fitting model for the data. Phylogeny was then inferred using RAxML 8.2.12 with the best fitting model and 1,000 bootstrap replicates. Scale bar indicates changes per site. To the right of each species are illustrations of the host(s) that each species has been reported to infect. This phylogenetic tree is largely in agreement with recent previously published phylogenies [3,4,8,9,19,23,27], with the exception of *Anncaliia algerae* or *Edhazardia aedis*, which have different placements in several reports [3,8,23,27]. (B) Comparision of protein content for each species. (C) Comparision of genome size for each species. (D) Morphological and molecular characteristics of each species. E-value, expect value; TOR, target of rapamycin.

Because of the intimate relationship between microsporidia and their hosts, they are heavily dependent on host resources and have undergone extensive genomic reduction [6,7]. Analysis of sequenced microsporidia genomes (Fig 1A) has revealed a wide loss of protein families that are present in other eukaryotes, leaving only approximately 800 conserved microsporidia proteins [6]. The proteins that have been retained function in essential core cellular processes such as DNA replication, transcription, and translation [6,8,9]. Proteins that have been lost include many metabolic enzymes, regulatory pathways, and proteins involved in vesicular transport such as TOR (Target of Rapamycin) and clathrin [8–10]. In addition, the proteins that are retained are shorter than their fungal orthologs, with *Encephalitozoon cuniculi* proteins being on average only 85% as long as their yeast counterparts [3,11]. One such instance of proteins that have decreased in size are the aminoacyl-tRNA synthetases, which have lost many regulatory regions, including a domain in leucyl-tRNA synthetase (LeuRS) that edits mischarged tRNAs. The microsporidia *Vavraia culicis* was shown to have mistranslation error rates of 5.9% at positions that coded for leucine—although valine, which has a corresponding tRNA synthetases with an intact editing domain, has a mistranslation rate of 7.5%, suggesting that there are other factors responsible for the high levels of mistranslated proteins [12]. Microsporidia also have reduced noncoding RNAs, including the 18s RNA, which is only approximately 2/3 as large as in other eukaryotes and results in the smallest eukaryotic ribosomes [13]. Although this reduction has led to several binding sites being eliminated, structural and mass spectrometry analysis of ribosomes from *Vairimorpha necatrix* demonstrated that most conserved ribosomal proteins can still bind [14]. Microsporidia also have reduced organelles, including mitochondrial remnants known as mitosomes [15]. These mitosomes do not encode a mitochondrial genome and are incapable of carrying out oxidative phosphorylation; microsporidia must instead import ATP from their host [16]. Interestingly, there are 32 conserved protein families in microsporidia that are not found in any other eukaryote [6]. Several of these proteins function as part of the polar tube or spore wall, and the function of most of these proteins is unknown [6].

## How do microsporidia genomes differ from one another?

Microsporidia genome sizes vary significantly. The largest is *Edhazardia aedis* at 51.3 Mb (encoding approximately 4,200 proteins), and *Encephalitozoon* genomes are the smallest, with some being only 2.3 MB (encoding approximately 1,800 proteins) [17] (Fig 1B and 1C). Much of this size disparity results from noncoding DNA, with only 9% of the *E*. *aedis* genome coding for proteins, compared to approximately 90% for *E*. *cuniculi*. The regions between genes can become very compact, with the intergenic regions averaging only 115 base pairs in *Encephalitozoon intestinalis* [17,18]. Though microsporidia have much fewer proteins overall compared to other eukaryotes, genomic reduction was followed by a large expansion in lineage-specific protein families [6]. For example, *Nematocida parisii* comprises 2,661 proteins, of which only 1,074 are conserved with other eukaryotes or with other microsporidia species outside of its genus. This means that over half of the proteins in *N*. *parisii* are unique to the *Nematocida* genus, with 318 not even present in *N*. *ironsii*, a closely related sister species [19]. Many of these unique genes are found in subtelomeric regions and likely arose through local duplication events [19]. Additional mechanisms that have changed the protein content of microsporidia species include whole-genome duplications and genes arising from noncoding regions [20].

Several key eukaryotic processes have been lost in some, but not all, microsporidia. The ability to splice mRNA transcripts has been lost multiple times in microsporidia evolution, including in *E*. *aedis*, *Nematocida* species, and *Enterocytozoon* species, which no longer contain functional spliceosome machinery [21] (Fig 1D). This has resulted in some genomes having no detectable introns and the loss of most conserved splicing proteins [3]. Although many microsporidia genomes have retained Dicer and Argonaute, the two key effectors of the RNA interference (RNAi) pathway, both of these proteins have been lost several times, including in all of the species in *Nematocida* and *Encephalitozoon* [4,22]. Although all microsporidia species have lost many metabolic enzymes, there are important distinctions such as differential losses in lipid biosynthesis genes [3]. Additionally, *Enterocytozoon* species have lost genes necessary for glycolysis, leaving them completely dependent on their host for energy production [23].

## What did the last common ancestor of microsporidia look like?

Microsporidia were once considered the earliest diverging eukaryotes, but sequencing of a large number of microsporidia genomes in the last several decades has made it clear that they belong to a group of the earliest diverging fungi [2,24]. More recently, the discovery and subsequent genome sequencing of related basal species has shed light on the evolutionary origin of microsporidia. Microsporidia are closely related to a group of obligate intracellular parasites called the Cryptomycota or Rozellomycota [25] (Fig 1A). The only one of these species to have its genome sequenced so far, *Rozella allomycis*, infects water mold using motile flagellated spores [26]. Different than microsporidia, *R*. *allomycis* possesses mitochondria and encodes a much larger set of proteins that is more conserved with other eukaryotes. To date, the two most early diverging microsporidian species that have been sequenced are *Paramicrosporidium saccamoebae (*classified as a Rozellomycota), an intranuclear parasite of amoeba, and *Mitosporidium daphniae (*classified as a microsporidia*)*, which infects daphnia [27,28]. Although there has been debate about whether these species should be classified as microsporidia, recent analysis of environmental samples supports the existence of an expanded microsporidia that includes a diversity of early diverging species [29]. *P*. *Saccamoebae* and *M*. *daphniae* differ from *R*. *allomycis* in that their spores have lost flagella and contain a polar filament, although invasion by *P*. *saccamoebae* is carried out through host phagocytosis [30]. While *P*. *saccamoebae* and *M*. *daphnia* have a reduced gene set compared to other fungi, they have undergone less genome reduction than canonical microsporidia. Both species also have mitochondrial genomes, although *P*. *saccamoebae* has a conserved electron-transport chain whereas both *R*. *allomycis* and *M*. *daphnia* have lost Complex I and have different nuclear-encoded genes that are thought to facilitate ATP generation [26–28]. Additionally, *P*. *saccamoebae* has more genes in common with distantly related fungi than with *R*. *allomycis* or *M*. *daphnia*, suggesting that these species have undergone independent gene loss during coevolution with their hosts [27].

Another early diverging group of microsporidia are the metchnikovellids, which parasitize apicomplexan gregarines. Recent genome sequencing of two of these species, *Metchnikovella incurvata* and *Amphiamblys* sp., has revealed that this group is most similar to the canonical microsporidia, as these species have undergone dramatic gene loss and do not contain mitochondrial genomes [8,9] (Fig 1). One of the most striking differences in metchnikovellids is the presence of clathrin, which has been lost in the canonical microsporidia [8,9]. Additionally, the 32 conserved protein families that were found to be specific to the canonical microsporidia are not present in *Amphiamblys* sp. [9], indicating that the canonical microsporidia have a number of proteins that differentiate themselves from the metchnikovellids.

Taken together, these genomic analyses suggest that the earliest steps in microsporidia evolution were the development of the defining polar filament and the loss of flagellum. This was followed by genomic reduction, including loss of the mitochondrial genome and subsequent expansion of microsporidia-specific and genus-specific gene families. Recent environmental sampling has revealed a great diversity of early diverging microsporidia species, and it is expected that additional whole-genome sequencing of these species will provide even greater clarity into the evolution of microsporidia [29]. Are there species that have evolved polar filaments while maintaining a flagella apparatus? Was the mitochondria genome lost in independent lineages? In species that have retained the mitochondrial genome, are there proteins besides Complex I that are commonly lost? Have other branches of early diverging microsporidia undergone as dramatic of gene reduction as in canonical microsporidia? Are there features associated with the transition to infecting animals? Was the last common ancestor of *Rozella* and microsporidia a parasite, or did this ancestor have a free-living lifestyle? Additional comparative studies of microsporidia and related species will help in answering these and other questions of how microsporidia evolved.

## What is the host specificity of microsporidia?

Microsporidia infections are extremely common in animal species. Fig 1A shows a variety of invertebrate and vertebrate species that microsporidia infect. Microsporidia also infect protists such as free-living ciliates and other parasites, including paramyxids, which infect bivalve mollusks [29]. Although most microsporidia have a narrow host range and infect only one or several closely related host species, several microsporidia species are generalists and have broad host ranges [3,31–33]. Examples of generalists include *Enterocytozoon bieneusi* and the *Encephalitozoon* species, which can infect a wide variety of birds and mammals [34]. Many genera of microsporidia show specialization to a certain group of animals, such as the nine known *Nematocida* species that parasitize nematodes [19,31,35]. Species within a clade have also been observed to have different host specificity. For example, *Encephalitozoon romaleae* infects grasshoppers, unlike the other vertebrate-infecting members of the genus. This suggests that host specificity can rapidly switch between vertebrates and insects [36]. There are also several examples in which microsporidia have independently evolved to infect the same species, including humans, mosquitos, and nematodes [31]. Interestingly, host ranges can be extended when microsporidia encounter immunocompromised hosts [37]. This is thought to be the case for several species that infect immunocompromised humans, such as *Trachipleistophora hominis*, whose natural host is thought to be an insect, and *Anncaliia algerae*, whose natural host is a mosquito [38,39]. Tissue specificities can also differ within a host [2]. Of two different species of *Nematocida* that infect *Caenorhabditis elegans*, *N*. *parisii* only infects the intestine, whereas *N*. *displodere* proliferates in several tissues including the muscle and epidermis. Differences in subcellular localization have also been observed, such as with the related crab-infecting species *Hepatospora eriocheir* and *Enterospora canceri*, which infect the cytoplasm and nuclei, respectively [23].

Although approximately 1,400 microsporidia species have been described, there is likely a large amount of undiscovered species diversity [7,40]. For example, a study sampling terrestrial nematodes identified 12 distinct microsporidia species, and *C*. *elegans* alone has been reported to be infected by seven different species [19,31,35]. Additionally, environmental sampling has identified dozens of novel species [41,42]. Based on the wide diversity of known animal hosts and the specificity of microsporidia species to infect one or several closely related hosts, it has been suggested that the number of microsporidia species equals the number of animal species. This has led to estimates of over 100 million microsporidia species [43].

### How have microsporidia adapted to their hosts?

Microsporidia use many proteins to directly interact with and manipulate their hosts. These host-exposed proteins contain targeting signals that direct them for secretion into the host or to the membrane of the parasite. Microsporidia species are predicted to have approximately 100 to 1,300 host-exposed proteins, which includes lineage-specific, expanded gene families [6,19]. These large gene families are particularly interesting as they can make up over 10% of a single microsporidia genome [35]. Although the function of these families is unknown, many family members contain protein–protein interaction domains, suggesting that they may interact with host proteins [19].

Because canonical microsporidia cannot undergo oxidative phosphorylation, they rely on their hosts for ATP and other metabolites. The most extensively studied microsporidia protein that directly interacts with hosts is hexokinase. Microsporidia hexokinases have gained a signal peptide and were experimentally shown to be secreted in several species [19,44–46]. This enzyme potentially increases host metabolism to provide nutrients for the developing parasites, and consistent with this idea, knockdown of the enzyme reduces microsporidia proliferation [44]. To obtain ATP from their hosts, microsporidia encode a nucleotide transport protein that was likely acquired via horizontal gene transfer from bacteria. These proteins allow for the transport of ATP into the parasite and have been diversified to acquire other substrates [16]. A second family of nucleotide transporters, the major facilitator superfamily, was recently shown to transport ATP and was found in many eukaryotes and in *Rozella* and all of the sequenced microsporidia [47]. Microsporidia can interact with host mitochondria, which is thought to increase the transport of ATP into the parasite. Recent work identified a protein from *Encephalitozoon hellem*, sporoplasm surface protein 1 (SSP1), that is on the sporoplasm that is involved in both invasion of host cells and association with the host mitochondria [48].

## Future perspective

Microsporidia were discovered over 160 years ago, but our understanding of these organisms has lagged compared to many other eukaryotic parasites of humans [24]. The use of genomic sequencing technologies has allowed for rapid advances in our understanding of how these cryptic parasites function and how they have evolved. Many other technologies are now being successfully applied to microsporidia. The application of whole-genome amplification to single infected host cells has allowed for the sequencing of species that are challenging to culture [8]. The use of ancestral gene reconstruction has revealed how nucleotide transporter genes evolved [16]. Proteomic technologies such as mass spectrometry have been used to study the localization of microsporidia proteins as well as their translational fidelity [12,14,19]. Cryo-electron microscopy has been used to determine the structures of microsporidia ribosomes. This work allowed the identification of a conserved, microsporidia-specific ribosomal protein (msL1) and also identified two conserved microsporidia dormancy factors, one that is conserved throughout eukaryotes (MDF1) and the other that was only found in several species of microsporidia (MDF2) [14]. Although it is currently not possible to genetically modify microsporidia, using RNAi to knockdown genes provides a powerful approach to directly study microsporidia protein function in the context of infection [44,49]. Finally, the discovery of microsporidia that infect model organisms such as *C*. *elegans* and *Drosophila melanogaster* provides easily cultured, genetically tractable hosts for studying how microsporidia function [2,19,35,50]. Continued use of the technological advances highlighted here is likely to provide additional insight into the function and evolution of these fascinating pathogens.
